# Literary Notes

**Published:** 1896-04

**Authors:** 


### Literary Notes.

### LAYS ON RAYS.

BY WILLIAM JAMES EVANS.

Turn on the Röntgen ray,
Let darkness have its day,
Crooke's tubes have come to stay,
Light shines the other way.

Let wit see through the dense
Old wooden door of sense,
With vision so intense
The field is quite immense.

When we've appendix fright
Can we then use the light,
And, finding it all right,
Not operate that night?

If the electric eye
Can see through wet and dry,
Inside both you and I,
What charms can it descry?

Will it heal the unsound,
And bring the sick around,
Make happiness abound,
And let those smile who frowned?

We'll pay our doctors' bills,
And put him in our wills,
If it bacilli kills
And cures our other ills.

Can we see who are right
With this new Röntgen light?
Will it help mental sight
To put the false to flight?

May it not see behind
Your eye into your mind,
And show what you're inclined,
To think no one can find?

It sees straight through a book,
It shames—the critics' look;
What you can't find by hook
Just radiograph by Crooke.

We hear a thousand miles,
And see through our profiles,
And now electric trials
Taste, smell and touch beguiles.

---

THE *Buffalo Sunday News*, of March 15, 1896, gives a clever review of a series of articles published in the March issue of the *American Journal of Medical Sciences* on the Röntgen X rays.

The *News* reproduces twelve illustrations from the journal mentioned and has done altogether the most scientific work on the new photography that we have seen in any newspaper.

---

WEIR'S index to the medical press is announced for issue about April 15, 1896. The object of this publication is to index the literature in the medical journals of the United States and Canada for the month previous, including also the published transactions of the various national and state medical societies. Although the "index" proper will deal strictly with the American medical press, British and European journals will be similarly reviewed in a voluminous appendix. Further, it is the intention of the management to list, under each department, recently-published text-books on the general subject, so that each issue may be, as nearly as possible, a complete bibliography of current medical literature. Subscribers to this journal may receive the initial number by addressing the publishers before April 15th, provided mention is made of the BUFFALO MEDICAL JOURNAL in the request. Address Frank Weir & Co., Publishers, 32 South street, New York.

---

A MAGAZINE'S INFLUENCE.—The enormous circulation of such a magazine as *The Ladies' Home Journal* can, in a sense, be understood when it is said that during the last six months of 1895 there were printed, sold and circulated over 4,000,000 copies—in exact figures, 4,058,891. Figures such as these give one some idea of the influence which may be exerted by even a single one of the modern magazines.

*The Ladies' Home Journal* does not accept medical advertisements of any sort. Every regular physician should testify his appreciation of this unique position taken by the editor, Mr. Edward W. Bok, by becoming a subscriber to it.

---

THE *Scalpel*, a monthly journal of medicine and surgery, edited by Dr. Thomas M. Dolan, Halifax, has appeared. It is announced that it will be continued on the same lines as the *Provincial Medical Journal*, which for ten years has been so well known under the same editor.

---

THE *Journal of Mechanical Surgery*, edited by Dr. Edward A. Tracy, Boston, who modestly announces himself as a member of the American Medical Association, is the latest candidate for professional favor. The object of this paper seems to be to advertise its editor.

The *Canada Medical Record* announces a change in its management. It is now owned and edited by the faculty of medicine of the University of Bishop's College, Montreal. Dr. J. B. McConnell is the editor.

